# Boiled or not boiled? – D-aspartic acid in bone proteins as proof of a burial “*more teutonico*”?

**DOI:** 10.1007/s00414-026-03928-5

**Published:** 2026-07-31

**Authors:** Melanie Schröder, Valerie Bühren, Alexandra Reckert, Julia Becker, Lisa König, Stefanie Ritz

**Affiliations:** Institute of Legal Medicine, Düsseldorf University Hospital, Düsseldorf, Germany

**Keywords:** More teutonico, Heating experiments, D-aspartic acid, Pentosidine, Bone proteins

## Abstract

Around the 12th/thirteenth century, a burial method called “*more teutonico*” was used for the remains of high-ranking individuals who had died far from their homeland and had to be brought back for burial. Before transportation, the bodies or body parts were boiled (macerated) to remove all soft tissue. The question of whether a body was boiled postmortem, is of particular interest for historians (burial “*more teutonico”*?) and this question may also be relevant in a forensic context, even if only in rare cases. In 1989, Bada, Herrmann, Payan and Man (“Amino acid razemization in bone and the boiling of Emperor Lothar I.” in Applied Geochemistry) suggested using the D-aspartic acid content of bone as an objective indicator of a postmortem exposure to heat. This approach was never substantiated by further studies, and more recent findings call the concept into question.

Therefore, we tested the “Bada approach” by analysing the D-aspartic acid content in boiled bone samples (pig, human) using different “boiling recipes” of procedures “*more teutonico*” (using water, wine, or vinegar as boiling media), and discussed it under consideration of the recent literature. Using the boiled human bone samples, we also investigated whether the question of “boiled or not boiled?” could be answered by another parameter, namely by analysing the pentosidine content of a sample.

An accumulation of D-aspartic acid was observed during boiling in both pig and human bone samples. It was considerably lower in the human samples and significantly influenced by the medium used. By contrast, boiling did not result in pentosidine accumulation.

These experimental results call into question the reliability of the “Bada approach” – not least because the type of boiling medium may not be recreated in each individual case. Moreover, this approach does not take the current knowledge on the intravital accumulation of D-aspartic acid in bone samples of different origins into account, nor the influence of postmortem conditions on the D-aspartic acid content in non-heated samples (used as controls by Bada et al.). Overall, the approach of Bada et al. should not be accepted uncritically, as proving a treatment “*more teutonico*” based solely on the D-aspartic acid content seems hardly possible – and if so, only under very strict conditions. The analysis of pentosidine is not suitable for demonstrating a burial “*more teutonico”*.

## Introduction

According to medieval documents, a burial method known as “*more teutonico*” (“in the German manner”) was used around the twelfth and thirteenth centuries for the remains of high-ranking individuals who had passed away far from home and had to be transported back for burial. Transporting bodies hundreds of kilometres was hardly feasible – not least because of the increasing putrefaction of the body. Therefore, the bodies were prepared for their transport according to the “*more teutonico”* method:

„Teutonici autem eviscerant corpora excellentium virorum, qui moriuntur in provinciis alienis, et reliqua membra tamdiu faciunt in caldariis decoqui, donec tota caro, nervi et cartilagines ab ossibus separantur, et postmodum eadem ossa, in odorifero vino lota et aspersa pigmentis, ad patriam suam deportant.“

“The Germans remove the entrails from the bodies of high-ranking men when they die in foreign lands and boil the remains in cauldrons until all the flesh, tendons, and cartilage are separated from the bones. These bones, washed in fragrant wine and sprinkled with spices, are then taken back to their homeland.” (Boncompagno da Signa Antiqua rhetorica 1215–1226, cited according to [1]).

According to the available sources, various procedures were used in the maceration process, which is what the burial *“more teutonico”* was; for example, the body was (or parts of the body were) boiled in water, vinegar or wine [1–3]. Many deceased, including Emperor Frederick I (“Barbarossa”; + 1190) [4], Isabella of Aragon (Queen of France; + 1271) [1] and Gerdag, Bishop of Hildesheim (+ 992) [5], were subjected to this procedure up until the year 1300, when Pope Boniface VIII banned this practice. Objective evidence of a burial *“more teutonico”* is of particular interest especially to historians. Even in a forensic context, the question of whether or not a body or body parts were heated postmortem may be relevant, e.g., when there is a suspected attempt to dispose body parts by “boiling”, or when body parts have been boiled in cases of cannibalism [6–8]. Although such cases are rare, the issue can play a significant role in individual instances.

Bada et al. [9] proposed using the D-aspartic acid (D-Asp) content of bone as an objective indicator of postmortem exposure to heat. The underlying argumentation was based on the assumptions that (1.) the non-enzymatic conversion of L-Asp into D-Asp is a temperature-dependent process and, therefore, (2.) boiled tissue should have a higher D-Asp content than non-boiled control samples. Based on this hypothesis, Bada et al. [9] examined bones of Emperor Lothar I. to determine whether he had undergone the procedure of a burial “*more teutonico*”. D-Asp levels in Lothar I’s bones were significantly higher than the values in bone samples of two other individuals (control samples) who had died at the same time but whose bodies were definitely not exposed to heat. These findings were interpreted as proof that Lothar I.'s bones had been exposed to considerable heat, meaning that his body had been boiled as part of a burial “*more teutonico*”. Based on data from experiments with deer bones, it was further deduced that Lothar I.’s bones had been boiled for 6 h ± 30 min [9].

A museum asked us to apply Bada’s approach to determine whether a historically significant individual whose skeleton was in their care had undergone the procedure “*more teutonico”*. They made this assumption because the individual died in medieval times in Italy and was buried in northern Germany, meaning that his body must have been transported hundreds of kilometres across the Alps. This request prompted us to take a closer look at the “Bada approach” for objectifying a postmortem heating (boiling) process.

This approach was never substantiated by further studies and, to our knowledge, has never been used again. Based on current knowledge, we assumed that the approach proposed by Bada et al. [9] underestimates the complexity of parameters affecting the D-Asp content in bone: D-Asp accumulates intravitally in bone [10–13], the D-Asp content of bone varies in samples from different regions of the skeleton [14], and other postmortem influences besides temperature can affect the D-Asp content of bones [15, 16].

Therefore, we tested the “Bada approach” experimentally by heating bone samples (pig, human) using different “boiling recipes” of procedures “*more teutonico*”, as described in the medieval literature. We also considered it worthwhile to conduct such examinations within a forensic context. In fact, we received occasional inquiries regarding whether we have any idea to determine whether bones or teeth samples had been exposed to heat after death.

Not least because this issue is also of forensic significance, we extended our investigations to include pentosidine as an additional parameter that might be suitable for detecting post-mortem heat exposure. This “Pen approach” is based on two underlying assumptions: (1.) Pen is an advanced glycation end product (AGE) that forms as part of the non-enzymatic Maillard reaction between reactive carbonyl species derived from sugars and lysine/arginine residues in proteins such as collagen [17]. In food chemistry, this process is accelerated in protein-rich matrices by heating, which promotes the formation of AGEs, including Pen, thereby contributing to heat-induced browning [18]. By analogy, one might hypothesise that postmortem boiling of bone samples could increase their Pen content compared to unheated controls. (2.) Because Pen is a relatively stable cross link in mineralised collagen [17] and has been shown to persist in historical samples [19], it should be possible to examine non-boiled material as control samples from the assumed time of death.

## Material and methods

### Experiments to estimate possible “boiling times”

Boiling experiments were conducted on 16 pig forelegs, obtained from a butcher, to estimate the boiling times required to separate the soft tissue from the bones. The pigs were between six and nine months old, which is the customary age for slaughtering fattening pigs.

Four pig samples were not boiled (control samples). The 12 remaining samples (four in each group) were boiled in water, white wine and vinegar (5% acid), respectively. Before boiling, all of the skin and most of the muscle tissue were removed manually using a scalpel and tweezers.

Each sample was placed in a stainless-steel pot containing the respective boiling medium and boiled on a conventional hotplate under observation. The boiling time was documented, and boiling was stopped as soon as all the soft tissue had detached from the bones.

After boiling, a piece of bone measuring approximately 4 cm × 2 cm × 1.5 cm was removed from each of the 16 pig cubits from the extensor area near the joint, below the olecranon at the level of the coracoid process using an oscillating saw.

### Boiling experiments on human skull samples

Skull samples were taken from the left parietal bone of five individuals with known ages between 23 and 43 years using an oscillating saw. Samples measured approximately 5 cm × 3 cm × 0.5 cm. The soft tissue was carefully removed using a scalpel and tweezers.

Each sample was cut into four fragments of approximately equal size. One portion of each sample remained non-boiled. The remaining three fragments of each sample were placed in commercial tea infusers and heated in a stainless-steel pot containing the respective boiling medium, under observation on a hotplate. Fluids were supplemented as needed. After boiling 5.5 h in water, 5 h in wine and 4.5 h in vinegar (boiling times according the results of the boiling experiments with pig bones, see below), the samples were removed from the media.

### Further processing of the pig and human samples

Any remaining soft tissue was removed using a Dremel multitool. The bone samples were then sawn into smaller bone fragments and pulverised using an IKA Tube Mill Control (17,000 rpm). The resulting powder was successively washed in distilled water, 15% sodium chloride, 2% sodium dodecyl sulphate and ethanol/ether (vol. 3:1), then freeze-dried and stored at − 20 °C until further analysis.

The D-Asp content was determined in the pig bone samples, while in the human bone samples both the D-Asp and the Pen content were determined. Pen content in pig bones could not be determined due to their young ages at death.

### Analytics

#### D-Asp content of pig and human bone samples

A small amount of bone powder from each sample was hydrolysed for 6 h with 1 mL of 6 N HCl at 100 °C. D-aspartic acid and L-aspartic acid were analysed using high-performance liquid chromatography (HPLC; 1100 Series and 1260 Infinity II, Agilent, CA, USA) as described by Becker et al. [20]. Samples were dissolved in 1 mL sample buffer (0.01 M HCl with 1.5 mM sodium azide and 0.03 mM L-homo-arginine). A C18 column (Hypersil BDS C18, 250 × 3 mm, particle size 5 μm, Thermo Fisher Scientific (TFS), Waltham, MA, USA) was used as stationary phase; for the mobile phase eluents A (23 mM sodium acetate, 1.5 mM sodium azide, and 1 mM EDTA) and B (92.3% methanol, 7.7% acetonitrile) were used. The amino acid enantiomers were detected by a gradient over a period of 49 min at a constant flow rate of 0.56 mL/min. D- aspartic acid (D) and L-aspartic acid (L) were identified by their retention times. The total content of D-Asp of each sample was expressed as ln((1 + D/L)/(1 – D/L)).

#### Pentosidine content of human bone samples

Approximately 40 mg of bone powder were hydrolysed with 1 mL of 6 N HCl at 110 °C for 18 h. The dried samples were dissolved in 200 µL of HPLC-grade water. Analysis was performed by HPLC (1100 Series). A semi-preparative column (Onyx™ Monolithic SemiPrep C18, 100 × 4.6 mm, Phenomenex, Torrance, CA, USA) was the stationary phase. The mobile phase consisted of eluents A (40 mM NaH_2_PO_4_, 1.5 mM sodium azide, 0.1% HFBA, pH = 2.70), B (45% methanol, 45% acetonitrile, 10% H_2_O) and C (40 mM NaH_2_PO_4_, 1.5 mM sodium azide, pH = 2.70) based on Heems et al. [21]. Samples were detected over a period of 39 min using a linear gradient. Eluent A was progressively replaced by the HFBA‑free eluent C, reaching 30% eluent C at 22 min. The flow rate was kept at a constant 1 mL/min and the column temperature at 40 °C. Pen was identified by its retention time. A calibration curve was used for calculating concentrations.

### Evaluation

The D-Asp and Pen contents of the boiled human samples were interpreted taking into account available datasets on the intravital accumulation of parameters in non-boiled samples (data from [20]).

## Results

**Fig. 1:**
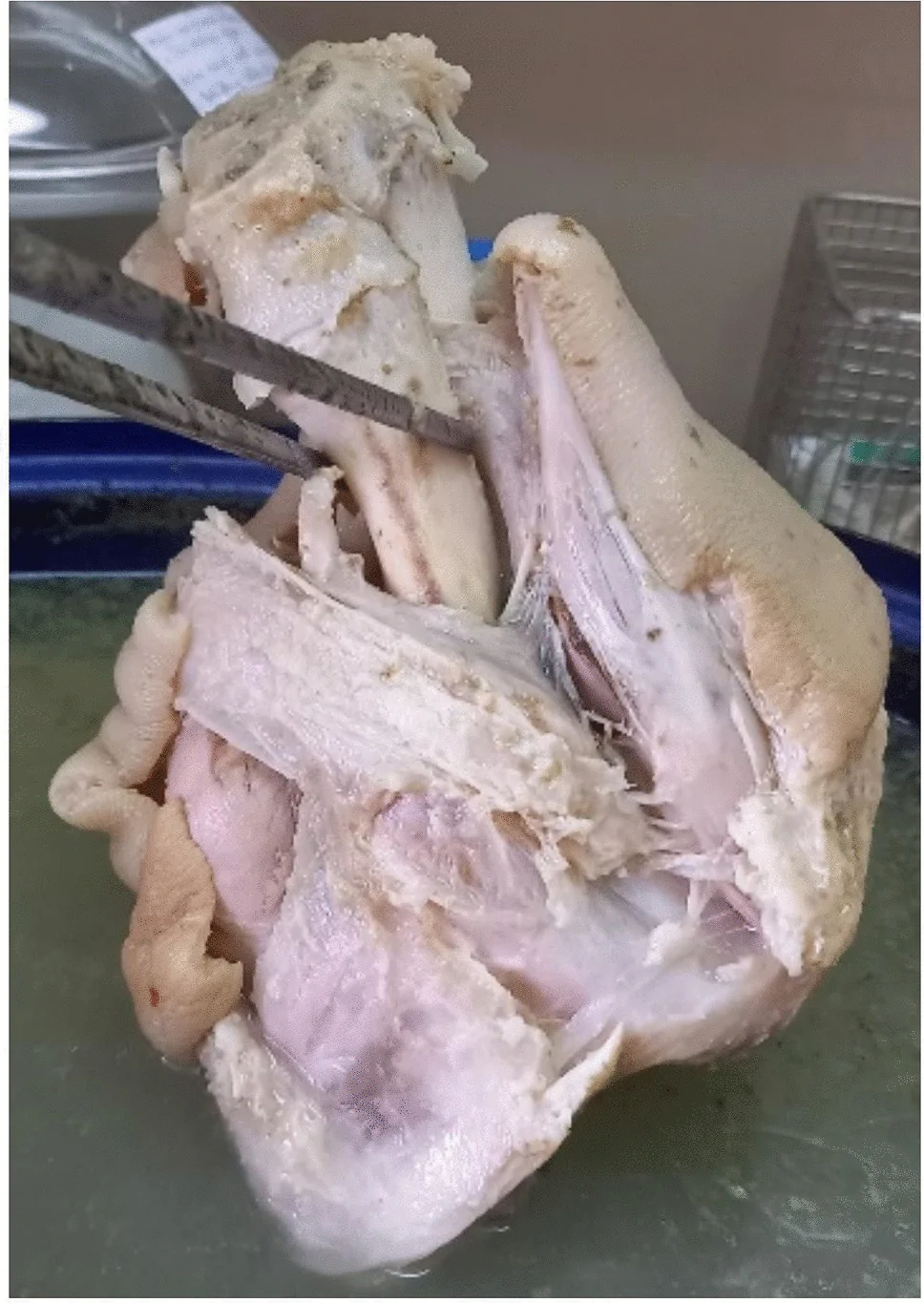
Pig sample after boiling “until all the flesh, tendons and cartilage are separated from the bones” [1] – Boiling time up to 5.5 h in water, 5 h in wine, and 4.5 h in vinegar

After boiling for up to 5.5 h in water, 5 h in wine and 4.5 h in vinegar, all soft tissues had detached from the pig bones (Fig. 1). The maximum “boiling times” were used in the subsequent boiling experiments (as described above).

### Boiling experiments – pig bone

**Fig. 2:**
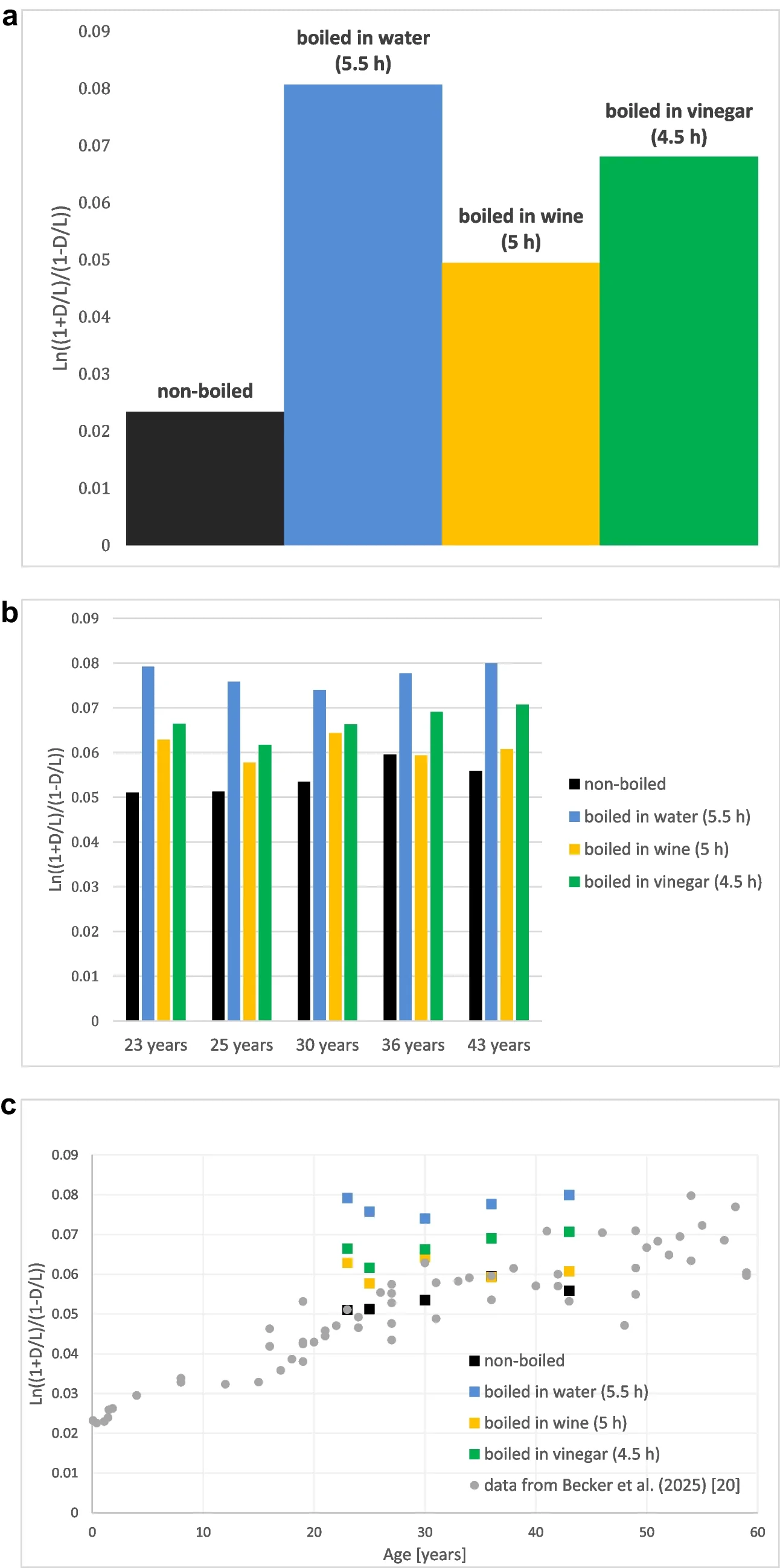
**a** Boiling experiment – pig bone/D-Asp. **b** Boiling experiment – human bone (skull)/D-Asp (samples from 5 individuals with different ages at death). **c** D-Asp values in human skull samples from the boiling experiments (5 individuals) in relation to previously published data from non-boiled skull samples (grey dots, from [20])

The D-Asp content increased significantly after boiling (increase of 111,56%–245,35% compared to the values of the non-boiled samples); the greatest effect was observed when boiling in water with a maximum increase to approximately four times the initial value for the non-boiled samples (Fig. 2a).

### Boiling experiments – human bone

As for the human samples, D-Asp levels were higher after boiling as well (Fig. 2b). The increase of D-Asp levels was lower than in the pig samples (increase of up to 55,14% of the values of the non-boiled samples). Again, the choice of the boiling medium had an effect, with the highest increase in D-Asp levels after boiling in water.

Figure 2c presents the D-Asp values in human skull samples from the boiling experiments in relation to previously published data from non-boiled skull samples (from [20]). D-Asp values after boiling in water are clearly beyond the range of variation in non-boiled samples; by contrast, the values after boiling in wine and vinegar are occasionally within this range of variation.

**Fig. 3:**
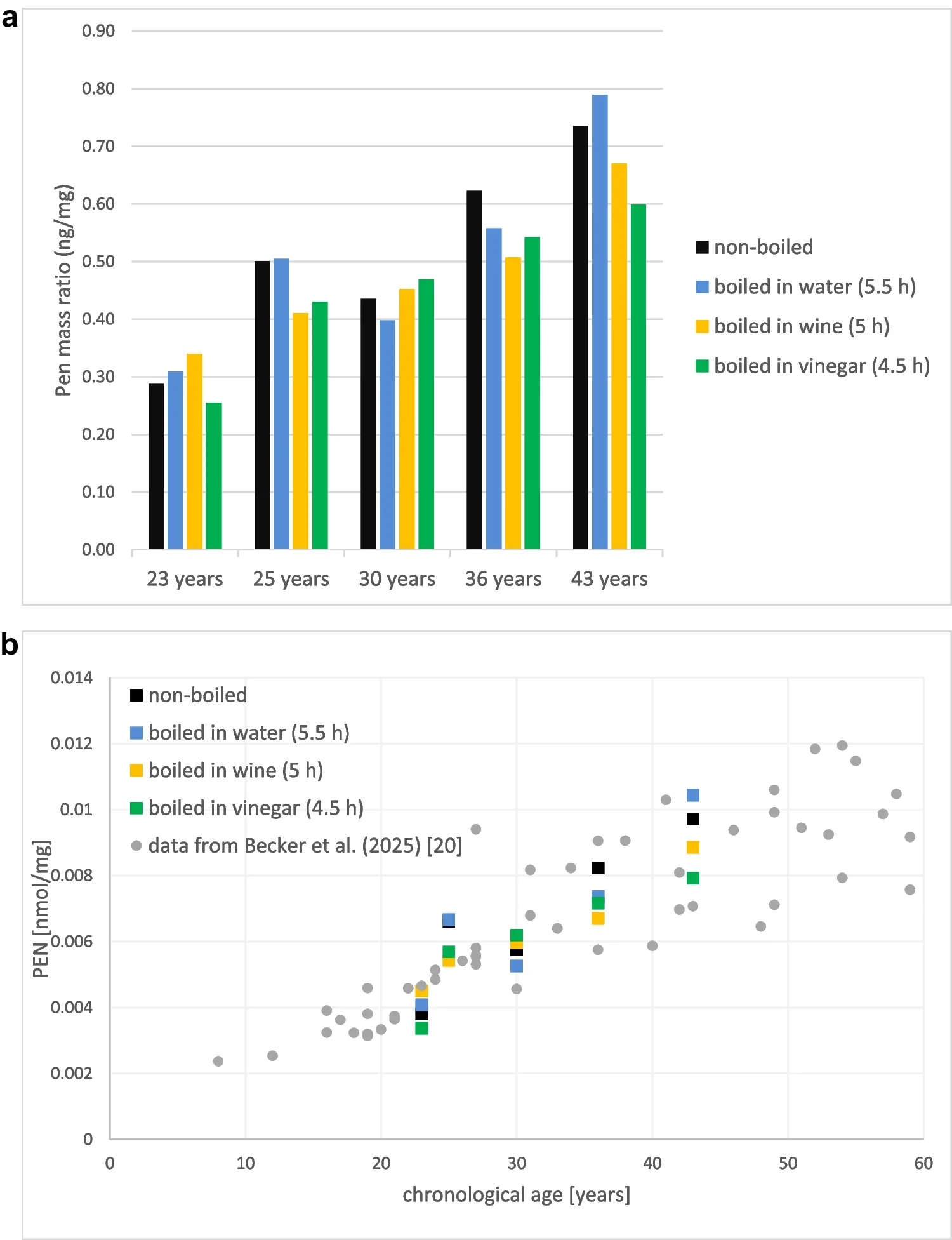
**a** Boiling experiment – human bone (skull)/Pen (samples from 5 individuals with different ages at death). **b** Pen values in human skull samples (5 individuals) from the boiling experiments in relation to previously published data from non-boiled skull samples (grey dots, from [20])

Contrary to D-Asp, Pen values after boiling scatter unsystematically around the value of the non-boiled samples (Fig. 3a). The Pen data do not exhibit substantive differences between boiled and non-boiled samples and lie within the range of non-boiled samples from individuals of corresponding ages (Fig. 3b).

## Discussion

Bada et al. [9] proposed to use the D-Asp content in bone to detect a postmortem heating of bone (as proof of burial “*more teutonico*”). Since this question can also be of significance in a forensic context, we reviewed this approach. We also tested whether postmortem boiling could be proven by analysing the Pen content of bone samples (“Pen approach”).

The number of human samples analysed is relatively small. After evaluation of the data presented here, it was very clear that no further significant insights were to be expected from analysing more human samples. Therefore—and ultimately for ethical reasons as well—it was decided not to analyse further human samples.

The “Pen approach” is not suitable to answer the question of whether bones had been heated (boiled) postmortem.

Pen proved to be remarkably heat-stable. In the boiling experiments, the Pen content of bone samples did not show significant differences between boiled and non-boiled samples (Fig. 3a, b). Therefore, the analysis of Pen is not suitable for proving a burial “*more teutonico”*.

The approach taken by Bada et al. [9] to clarify the question of a burial “more teutonico” should not be used uncritically.

The approach proposed by Bada et al. [9] is based on the basic assumption that temperature determines the D-Asp content of bones so significantly that an increased D-Asp content in a bone sample can be considered as proof of heat exposure. Unfortunately, this basic assumption cannot be maintained in view of the recent literature and our experiments, which demonstrate a high complexity of the factors determining the postmortem D-Asp content in bone, which were not considered by Bada et al. [9].

### Water is not wine: The boiling medium influences the kinetics of D-Asp accumulation in bone

Bada et al. [9] assumed that Lothar I was boiled in water. However, this may not have been the case, as historical sources also suggest that wine and vinegar were used for boiling of human remains. Our boiling experiments demonstrate a significant influence of the type of the boiling medium used (Fig. 2b, c). This is likely due to different effects on the inorganic and organic bone matrix. Different influences from boiling media may lead to different degradation processes and molecular changes [22–24]. This effectively changes the protein composition of the samples. Since the kinetics of the conversion of L-aspartic acid to its D-form depend on the structure of the respective “parent proteins” [24–26], this may result in altered kinetics of the heat-induced accumulation of D-Asp. As it is likely impossible to reconstruct the medium used for boiling in most (historic) cases, it will remain unclear to what extent the boiling process could have influenced the D-Asp content of a bone sample.

### The selection of control samples is critical, but achieving this optimally is difficult

For the “Bada approach” it is of central importance to compare the D-Asp content of presumably boiled samples with the D-Asp content of non-boiled samples from individuals with a comparable postmortem interval. However, when selecting their control cases, Bada et al. [9] failed to consider some relevant findings:

Bada et al. [9] did not consider the intravital accumulation of D-Asp in bone tissue (see data for non-boiled samples in Fig. 2c and [10–13]). The age at death significantly determines the D-Asp content in bone. The intravital age-dependent accumulation of D-Asp results in different initial values at the time of death, before any further increase in D-Asp content may have been provoked by heat exposure after death. Bada et al. [9] did not state the age at death of their control individuals. If these two individuals were significantly younger than Lothar I. when they died, this could explain significantly lower D-Asp levels in their bone samples, at least to some extent. In any case, Bada et al. [9] should have considered the intravital “basal accumulation” of D-Asp and should ideally have used non-boiled control samples from individuals of a similar age at death.

Moreover, Bada et al. [9] obviously did not consider the structural and metabolic differences of bone at specific anatomical sites, resulting in different kinetics of the intravital accumulation of D-Asp [14]. When applying the approach of Bada et al. [9], the sample to be tested would need to have come from the same anatomical location as the control samples. Bada et al. [9] do not provide any information on the anatomical region of the samples they analysed.

A further point not considered by Bada et al. [9] is the possibility of increased D-Asp content in non-boiled samples due to long postmortem intervals. Depending on postmortem conditions, D-Asp may accumulate during the postmortem interval, even in the absence of heat exposure. Other conditions apart from high temperature that can lead to an unusually rapid degradation of bone proteins are also relevant, such as specific microbial influences or exposure to high humidity, and/or extreme pH conditions [15, 27–30]. Protein degradation during the postmortem interval may result in small peptides with only low steric hindrances and a significantly faster formation of D-Asp residues [24–26]. Mahlke et al. [19] report unusually high D-Asp contents in (non-boiled) dentine in cases with long postmortem intervals and discuss a postmortem increase of D-Asp due to degradation. Without knowledge of the environmental conditions during the entire postmortem interval, the interpretation of strikingly high postmortem D-Asp levels is difficult, especially the differentiation between the effects of heat exposure and those of other degradation-inducing influences.

### The kinetics of the heat induced accumulation of D-aspartic acid in animal bones are not comparable to those in human bones

Using data from boiling experiments with deer femur bones, Bada et al. [9] came to the conclusion that Lothar's bones were boiled for “6 h ± 30 min”. However, our boiling experiments with pig and human bones revealed that the data obtained from animal bones cannot easily be translated to human bones. The pig bone samples show a significantly faster accumulation of D-aspartic acid at identical heat exposure. This could be due to lower steric hindrances and fewer crosslinks in the collagen, especially since the pigs were very young at the time of death (6–9 months). Dobberstein et al. [31] reported very similar results from heating experiments with teeth: The dentinal collagen from young pigs degraded much faster than that from humans (16–28 years old).

*All things considered, the proof of a treatment “more teutonico” based solely on the “Bada approach” and the D-aspartic acid content seems hardly possible – and if so, only under very strict conditions.*

If, despite all concerns, the use of the method proposed by Bada et al. [9] should nevertheless be considered, the following prerequisites must be met: It should be examined whether there is any information (e.g., from historical sources) regarding the “boiling method” used in the specific case. The non-boiled/unheated control samples should come from individuals with similar or, ideally, identical ages at death and death dates, and they should also stem from identical anatomical locations. Furthermore, it should be ensured that all samples show no evidence of unusual influences during the postmortem interval.

## Data Availability

The data of this study are available from the corresponding author upon request.

## Declarations

In partial fulfillment of the requirements for a doctoral degree.
